# The Effect of “In Favor of Myself”: Preventive Program to Enhance Positive Self and Body Image among Adolescents

**DOI:** 10.1371/journal.pone.0078223

**Published:** 2013-11-12

**Authors:** Moria Golan, Noaa Hagay, Snait Tamir

**Affiliations:** 1 Shahaf, Community Services for the Management of Weight-Related Problems, Tel Aviv, Israel; 2 Department of Nutrition, Tel Hai Academic College, Upper Galilee, Israel; 3 School of Nutritional Sciences, The Hebrew University of Jerusalem, Rehovot, Israel; Universidad Europea de Madrid, Spain

## Abstract

**Background:**

Positive self-esteem, emotional well-being, school achievements and family connectedness are considered protective factors against health-compromising behaviors.

This study examined the effect of an interactive, community-based, media literacy and dissonance wellness program, *In Favor of Myself*, on the self-image, body image, eating attitudes and behavior of young adolescents. A preliminary cohort study was conducted among 972 program participants who did not take part in the controlled trial. Over 75% of participants said they would recommend the program to their friends.

**Methods:**

A controlled trial was conducted to evaluate program acceptability, efficacy and effectiveness among 259 participants (210 in the intervention group and 49 in the control group), aged 12–14 years, who completed questionnaires during at least two assessment times. Program materials were provided, along with leaders' training, in order to ensure quality program delivery and creation of a wide network of committed program leaders.

**Results:**

The program significantly reduced drive for thinness and self-worth contingent upon others' approval, the gap between current body figure and ideal figure, and the impact of mood on girls' self-image. Superiority was found among those participating in the intervention group with respect to recognizing media strategies, the influence of media on desire to change, and the influence of appearance on self-confidence and drive for thinness.

**Conclusions:**

In Favor of Myself shows promising results for strengthening adolescents' ability to cope with the challenges of their life stage. Suggestions for improving In Favor of Myself are presented.

## Introduction

Adolescence is a critical life stage for the development of self-confidence and self-image [1]. Since puberty, by its very nature, is associated with weight gain, adolescents frequently experience dissatisfaction with their changing bodies. In a culture that glorifies thinness, some adolescents, mostly girls, become excessively preoccupied with their physical appearance and begin to diet obsessively in an effort to achieve or maintain a thin body. Socio-cultural agents, such as peers, parents, and the media are also hypothesized to contribute to lower self-esteem and development of eating disordered attitudes and behaviors [2], [3]

### Risky behaviors, well-being and self-image

Low self-esteem and negative self and body image have been reported as the most important factors for risk-taking behavior [4], [5]. Teenagers with low self-esteem often fall victim to a variety of unhealthy behaviors and may become more susceptible to peer pressure, while others may remain more self-centered. Furthermore, the desire to raise social status and impress peers leads many teenagers down a path of self-destruction [6].

There are three factors, known as prospective predictors, which are considered common to many adolescents and thus targeted in health promotion programs; these are: self-image, body image and self-esteem. Development of a healthy self-image is the result of self-image change, a process which occurs over a lifetime, and starts with learning self acceptance and being liked and accepted by others.

Body image is determined by a series of individual and socio-cultural factors common to Western society. Negative body image is a widespread concern among college age females and often results in dieting behaviors, which can perpetuate the risk of eating disorder development. Body image dissatisfaction is increasingly being recognized as an important target for public health action [7], [8].

The third factor, self-esteem, is a widely used concept which refers to an individual's sense of his or her value or worth, or the extent to which a person values, approves of, appreciates, prizes, or likes him or herself [9]. The vast majority of the research argues that self-esteem is positively associated with adaptive outcomes and health-related behaviors [10]–[12]. However, recent literature reviews suggest that self-esteem may not be the panacea it has been made out to be [4] and may be the result rather than the cause of improved academic performance [13]. Positive self-esteem has been proposed as an important defense against peer pressure and media influence, while low self-esteem is considered a risk factor that contributes to susceptibility to external influences, such as peer and media pressure.

### Effective health promotion programs

There is growing evidence that effective health promotion interventions for a specific risk or protective factor (a prospectively identified mediator or moderator of a risk process) are likely to have direct effects on a range of health outcomes [10], [14]. In order to cope with those risk factors that contribute to negative self and body image as well as risky behaviors, it is necessary to carry out a risk and protective factor approach to adolescent health promotion via social interventions, in order to change the personal motivation that can lead to such disorders.

According to Neumark-Sztainer et al [5], [10], since interventions promoting enhancement of global issues such as self-esteem, emotional well-being and social support may result in a decrease in some health-compromising behaviors, the main objectives of school-based prevention programs should be to identify and criticize the aesthetic beauty model, to develop critical thinking skills and to challenge the glorification of thinness for girls and muscular ideal for boys. Such programs aim to support good health through positive self and body image in different social contexts, without interferences associated with body image.

While most programs have focused on girls [5], a few universal prevention programs have focused on improvement in body image, knowledge about eating disorders, body satisfaction and self-image and reducing shape and weight concern of adolescents for both boys and girls [15]–[16]. Pratt & Woolfenden [17] did not arrive at firm conclusions in their review of the impact of eating disorder prevention programs for children and adolescents, none of the pooled comparisons indicated evidence of harm. Programs that take a more developmentally-oriented, affective approach, with a focus on increasing self-esteem and self-worth, decreasing feelings of alienation, accepting the different, and developing decision-making and positive communication skills have been suggested [17]–[19].

Factors that may influence the effectiveness of prevention programs include program format, participant risk status, age of participants, type of intervention and number of sessions [20], [21].

*In Favor of Myself is* an interactive (rather than educational) and universal program (?>rather targeted or universal-selective prevention program). It is delivered to mixed gender groups and aims to promote development of a healthy self-image and body image among adolescents to prevent later symptoms of hazardous behaviors (including eating problems). In contrast to other programs, its objectives are the enhancement of global issues such as promoting self-care, stronger ‘self’ and better immunization to harmful external influences (rather than focusing on preventing eating disorders), thus it was assessed among healthy early-adolescence participants. It is a multi-component program with multiple target areas, including positive self-esteem, communication, media literacy and dissonance health education, to promote a positive attitude towards growing up, sense of self and self-esteem, body image, and communication among adolescents. The program aims to promote the ability to filter external media and culturally inappropriate messages about diversity of beauty and attractiveness. The program includes structured supervision and has the potential for high generalization due to its wide dissemination across various setting (schools, youth movements, community settings) as well as different socio-economic groups. It takes into consideration the impact of ethnic background, religion and the intersection of gender on body image and eating problems [5].

## Methods

### Participants

**Figure 1:**
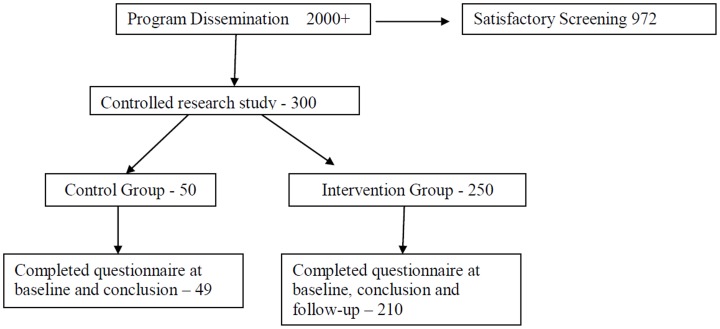
Participation flow.

Three hundred adolescents were recruited for the research study from the same grade level in a primary school with mixed gender and socio-economic populations (Figure 1). Two classes out of eleven were chosen arbitrarily to serve as controls (scheduled to receive the program the following year due to time-table constrains forced by the school management). Only 259 participants (210 in the research classes and 49 in the control classes), who filled out the questionnaire on at least two occasions, were analyzed for the evaluation of the program.

### Program description

*In Favor of Myself* is an innovative, interactive educational outreach program, developed and implemented by a group of professionals aiming to empower adolescents to promote their growth and development by teaching a range of coping strategies to help adolescents resist media-based and culturally inappropriate messages and promote the diversity of beauty and attractiveness.

### Program objectives

The program focuses on the promotion of adolescents' knowledge of normal development including the different courses of puberty among different groups, as well as the myriad of changes that take place during adolescence and their impact. It also aims to increase awareness of media strategies and literacy among youth as a way to help lower unrealistic expectations and decrease concerns with appearance. It teaches participants to identify and counter prejudice and socio-political structures that contribute to the development of body preoccupation. By helping youth gain the skills necessary to critically evaluate strong societal messages for both males and females vis-à-vis self-esteem, happiness, ideal body type or image, the program aims to help mitigate the effects of these messages.

### Program content

The program contains a kit with introductory material as well as a detailed guide for facilitators and material for eight interactive sessions (see enclosed photo). The guide includes eight structured sessions (Table 1) each of describe in detail, the background of the topic and offer interactive activities to engage participants both verbally and non-verbally. The kit also features a comprehensive introduction to a number of topics, including the consumer culture of Western society and its impact, adolescence as a period of transition, attractiveness and its various aspects, body image and self-esteem, and using art as a mirror of culture and as a space for self-expression. The guide uses inspiring and empowering techniques to strengthen body image and self-esteem such as employing dramatization that puts the beauty myth on trial and discuss the subject of attractiveness.

**Table 1:** Contents and description of sessions.

*Session number*	*Topics*	*Descriptions*
1	Adolescence, self esteem and positive interpersonal communication	Discussion about meaning of adolescence. Interactive activity about component of self-esteem, how they are developed and influenced by negative vs. positive communication. Positive communication ‘game’.
2	Filtering messages from the mass media advertisements	Analysis of advertising messages their interests and manipulations. Creating advertisement. Demonstration of Photoshop illusions.
3	Filtering messages from cultural origin stereotypes	Interactive activity and discussion about transmitted values delivered by famous figures and stereotypes
4	The beauty myth	Watching beauty in art throughout history and nations, discussion about changes in the criteria for beauty, the Real Women activity – respecting differences and variety of sizes. Putting the beauty myth on trial and questioning the automatic association between beauty and happiness.
5	The power of words, intrapersonal communication, body image and self esteem	Sharing internal stories about self esteem and body image. Exercising cognitive dissonance to challenge destructive perceptions and messages
6	The power of words, interpersonal communication and self esteem	Sharing the impact of negative interpersonal communication. Role play to challenge negative communication
7	Me, internally and externally.	Self insight by creating metaphor to ‘the self’ (internal and external). Sharing common and differences
8	Adopting a stance where to take it further	Summation of contents. Interactive activity regarding future stance

#### Satisfaction from the program

The satisfaction from the program was evaluated in a cohort study (performed in parallel to the controlled study) by an independent researcher with all adolescent who participated in the program during its first 6 months (972 subjects). Participants were asked to rate their satisfaction: 754 (77.8%) scored it as good or excellent, 91(9.3%) scored it as moderate and 127 (13%) did not like it.

### Leaders' Training

The program was delivered by teachers, school counselors and master program's students in nutrition – all supervised by the first author.

Leaders' training is provided to ensure quality program delivery and create a wide network of leaders who are committed to delivering the program. Each leader participated in a 6-hour introductory training which included 2-3 sessions. Such a participatory approach, as suggested by Neumark-Sztainer et al [5], is applied in order to increase the participants' sense of ownership and facilitate the sustainability of the intervention by providing topics and suggested activities for sessions, while encouraging the modification of games and their adjustment to suit the culture of participants. Adherence to program topics, spirit and key activities was assessed via audio taping of a sample of groups' interventions. In addition, an internet portal was developed to allow greater access to information by end-users and provide a venue for communication between participants (leaders and cadets) and experts.

### Procedures

The study protocol was approved by Tel Hai institutional review board. Parents of all participants, in the intervention and in the control groups, received information about the study and the intervention and provided a written informed consent. The program was delivered in a course of eight, 90-minute sessions, given approximately one week apart and integrated into a regular school coping skills curriculum. Groups of 15–20 participants were assigned 1 leader and groups of 21–35 participants were assigned two leaders. Twenty trainers and teachers were trained to deliver the program and the task of data collection was assigned to research students. Each participant was given an identification number to ensure confidentiality and anonymity.

### Assessment

The impact of the intervention was assessed with a non-randomized, controlled experimental group design. To assess the impact and outcome of the intervention, all participants in the research and control groups completed the study questionnaire at pre, post (8 weeks later) and 3-month (after post) follow-up assessments. Process evaluation, which assesses program dissemination, attendance and relapse rates, as well as assessment of participants' satisfaction, was performed at the end of the program.

#### Measures

Demographics, including personal and familial details, were obtained from each participant at baseline. Self-report measures are described in Table 2, all of which showing satisfactory levels of internal reliability. In order to extend the information received and assess the impact of positive and negative known predictors for health behaviors, 6 more questions were added to all these scales.

**Table 2:** Self-report measures' description.

Variable	Description of measures	Cronbach's internal consistency
Pressure by media	Sociocultural Attitudes towards Appearance Questionnaire-3 (SATAQ-3) – the media pressure subscale [29].	α = 0.96
Others' approval and appearance	Contingencies of Self Worth Scale (CSW) - others' approval and appearance subscales [30]	α = o.87 for others approval α = o.79 for appearance
Self-esteem	The Rosenberg Self-Esteem Scale (RSE) [31].	*α* = 0.90
Eating attitude and behaviors	Eating Attitudes Test-26 (EAT-26) [32].	*α* = 0.83
Drive for thinness and Body dissatisfaction	Eating Disorders Inventory -2 subscales (EDI-2) [33].	Drive for thinness *α* = 0.85 Body dissatisfaction *α* = 0.90

To assess program impact on knowledge and influence of media, participants were asked to identify several media strategies that promote consumption and internalization of stereotypes. They were also asked to rate media influence on their desire to “fix” their appearance. Further questions included use of comparisons to peers, family members and admired celebrities, as well as use frequency of positive and negative communication with peers and siblings. In order to evaluate change in awareness, perception of the changes occurring during adolescence and promote positive attitude towards them, subjects were asked to respond to the comment: “As I get older I feel that a) I am more influenced by the media b) life seems to be are more complicated c) life seems more interesting & exciting.” Test-retest evaluation was performed on the extended inventory with 30 students prior to administration of the questionnaire in order to assure its consistency with repeated completion (r = 0.87; p<0.01).

### Data Analysis

Participants were clustered in 9 program-groups. In order to take into consideration the unit treatment additivity the intraclass correlation coefficient (ICC) was calculated for each variable. Due to reasonable ICC (range of 0.03–0.07) the analyses were performed with all data set together.

One way analysis of variance (ANOVA) and chi-square tests were utilized to assess comparability of the groups on baseline measures. One way MANOVA with repeated measures and univariate ANOVA were used to assess program impact on participants in the intervention group by measuring the differences between baseline, post-intervention and three months follow-up.

2×3 MANOVA (groups X time) with repeated measure on time was used to assess the difference between groups in changes over time. The change in scores over time was analyzed after the intervention (pre->post) and over the 6 months study (pre->3 months follow-up).

Statistical analysis was conducted only for those who completed the questionnaire in at least two assessment times, including 210 participants in the intervention group and 49 participants in the control group (Total of 259 subjects). No differences were observed in baseline variables between those who filled the questionnaire on two or three occasions and those that did not (filled only on baseline).

Effect size is described using partial η^2^ (Partial eta-squared), where 0–0.05 constitutes a small effect, 0.059–0.1 a medium effect and above 0.10 a large effect [22]. Statistical analyses were undertaken using SPSS computer program (SPSS, Chicago, IL) for Windows; a p value of <0.05 was considered statistically significant.

## Results

### Baseline Characteristics of Participants

Table 3 shows the description of the studied population at baseline (mean age 13.5±1.0)

**Table 3:** Demographic description of studied population (% or mean±SD).

Variable		Intervention (n = 210)	Control (n = 49)	P
Age (years)		0.86±13.52	1.16±13.86	NS
Number of siblings		1.03±2.08	0.92±1.93	NS
Gender	Boys	45%	33%	P = 0.02
	Girls	55%	67%	
Country of birth	Israel	92.8%	91.8%	NS
	Other	7.2%	8.2%	
Family marital status	Parents married	87%	81%	NS
Father education	Academic degree	35.5%	38.5%	NS
	Vocational	25.5%		25.0%
	High school	39.0%	37.5%	
Mother education	Academic degree	52.2%	43.8%	NS
	Vocational	20.8%	29.2%	
	High school	25.0%	27.1%	

No statistically significant differences between the intervention and the control groups were revealed in the assessed variables, but there were many more girls (2-fold) than boys in the control group.

### Process Evaluation

The process evaluation with the controlled study population revealed that 78% evaluated the program as good or excellent while 12% did not like it. On a scale of 1 to 4, mean satisfaction with the program among participants was 2.93±0.9. 52% of participants attended all eight sessions, 14% attended 7 sessions, 18% attended 6 sessions, 16% attended 5 or fewer sessions.

### Intervention effect

The effect of the intervention on the following group of measures at conclusion and at 3 months follow-up, in comparison to baseline, is reported in Tables 4–6.

**Table 4:** Awareness of changes during adolescent and use of negative and positive communication.

	T_1_	T_2_	T_2_-T_1_			T_3_	T_3_-T_1_		
Measures	Baseline	Conclusion	F	P	Partial η2	Follow-up	F	P	Partial η2
Influenced by media	1.85±0.8	2.14±0.9	9.7	**0.02**	**0.08**	2.00±0.9	2.04	0.15	0.01
Awareness to life burdens	1.90±0.9	2.92±0.9	76.77	**0.00**	**0.4**	2.81±1.0	52.08	**0.00**	**0.31**
Life seems interesting	1.76±0.7	1.97±0.8	4.85	**0.03**	0.04	1.98±0.9	4.30	**0.04**	0.035
Positive communication	3.63±1.5	3.93±1.4	4.9	**0.02**	0.04	3.84±1.6	5.80	**0.017**	0.05
Negative communication	3.12±0.6	3.10±0.7	2.97	0.08	0.02	3.0 ±0.8	7.25	**0.008**	**0.06**

Note: Partial η2<0.05 is a small effect, 0.059–0.1 a medium effect and **above 0.1 large effect**. Paired comparisons' ANOVA: means, standard deviations and effect size (Partial eta-squared) on participants in the intervention group at base line, post- intervention and 3- month follow up.

**Table 5:** Media literacy, pressure from media and wish to fix appearance.

	T_1_	T_2_	T_2_-T_1_			T_3_	T_3_-T_1_		
	Baseline	Conclusion	F	P	Partial η2	Follow-up	F	P	Partial η2
**Number of media strategic identified**	4.75±1.38	5.24±1.34	10.4	0**.002**	**0.09**	5.03±1.3	3.63	**0.05**	0.03
**Pressure from media**	3.25±0.63	3.35±0.6	6.15	**0.01**	0.05	3.32±0.6	NS		
**Wish to fix appearance**	3.35±0.4	3.28±0.7	NS			3.1±0.7	4.23	**0.04**	0.03

Note:Partial η2<0.05 is a small effect, 0.059–0.1 a medium effect and **above 0.1 large effect**. Paired comparisons' ANOVA: means, standard deviations and effect size (Partial eta-squared) for participants in the intervention group at base line, post- intervention and 3- month follow up.

**Table 6:** Drive for thinness and self-worth.

	T_1_	T_2_	T_2_-T_1_			T_3_	T_3_-T_1_		
	Baseline	Conclusion	F	P	Partial η2	Follow-up	F	P	Partial η2
**Drive for thinness**	8.65±1.3	7.25±1.3	10.3	**0.002**	**0.09**	7.33±1.2	NS		
**Self-worth contingent upon others' approval**	2.59±0.6	2.05±0.5	69.7	**0.000**	**0.37**	2.09±0.5	50.2	**0.000**	**0.30**
**Self-worth contingent upon appearance**	2.30±0.5	2.14±0.5	11.00	**0.01**	**0.085**	2.19±0.5	2.9	0.09	0.02

Note: Partial η2<0.05 is a small effect, 0.059–0.1 a medium effect and **above 0.1 large effect**. Paired comparisons' ANOVA: means, standard deviations and effect size (Partial eta-squared) for participants in the intervention group at base line, post- intervention and 3- month follow up.

#### Knowledge about adolescence and communication with peers

One way MANOVA with repeated measure revealed significant difference between the three measure times in respect to the intervention group participants awareness of the myriad of changes that take place during adolescence [F(6,208) = 14.85, p<0.001, η2 = 0.44]. Univariate ANOVA revealed significant effect of time on the three measures [influence by media (F(2,208) = 3.8, p<0.02, η2 = 0.03;awareness to life burdens F(2,208) = 50.8, p<0.001, η2 = 0.303; life seems more interesting F(2,208) = 3.2, p<0.04, η2 = 0.03].

The means and standard deviations of each measure and the paired comparisons' ANOVA results are presented in Table 4. Participants in the intervention group reported (statistically significant) improvement in their attitude towards adolescence, noticing, “life seems more interesting and exciting” both post intervention and at follow-up (Table 4).

MANOVA also revealed significant difference between the three time frames in respect to the use of positive and negative communication with siblings and friends [F(4,201) = 8.10, p<0.01, η2 = 0.14]. Univariate ANOVA revealed significant effect of time on the two measures [(Positive F(2,201) = 8.8, p<0.001, η2 = 0.15; Negative F(2,201) = 5.8, p<0.01, η2 = 0.17 ]. Participants in the intervention group reported on increased use of positive communication skills, as well as a reduction in negative communication with peers (Table 4).

#### Media literacy, pressure from media and wish to fix appearance

One way MANOVA with repeated measure revealed significant effect of time on media literacy, pressure from media and the wish to fix appearance [F(6,204) = 6.7, p<0.01, η2 = 0.1]. Univariate ANOVA revealed significant difference between times in respect to media literacy (F(1,204) = 5.76, p<0.041, η2 = 0.05), pressure due to media influence (F(2,183) = 10.4; p = 0.002, η2 = 0.09) and effect on the wish to fix appearance [F(1,206) = 5.88, p<0.01, η2 = 0.04]). Paired comparison tests (Table 5) showed increase in participants' ability to recognize media strategies, engaged by publishers, to promote public consumption as well as less influence by the media pressures at post and at follow-up. A significant reduction was also noted with respect to “wish to fix appearance” with improvement reaching statistical significance only at follow-up (Table 5).

#### Drive for thinness, eating attitudes, self- worth and self esteem

MANOVA revealed significant differences between pre, post and follow-up in respect to eating disorders' features which were measured by EDI-2 subscales: drive for thinness and body dissatisfaction [F(4,203) = 4.39, p<0.01, η2 = 0.12]. Univariate ANOVA revealed significant effect of time only on drive for thinness [(F(2,208) = 3.8, p<0.04, η2 = 0.03] while body dissatisfaction (EDI-2) did not change significantly from baseline among program participants [(F(2,208) = 0.11, NS]. Paired comparison revealed that drive for thinness (EDI-2) decreased significantly among program participants, with a medium effect size of 0.09 at program conclusion and no further change at follow-up (Table 6).

EAT-26 scores indicated that the population had, a priori, a healthy mean score with respect to disordered eating and attitudes, thus it was not surprising that MANOVA revealed no significant differences among assessment times [(F(2,210) = 2.44, NS].

MANOVA revealed significant differences between assessment times in respect to participants' self-worth contingent upon others approval and upon appearance [F(4,206) = 20.04, p<0.0001, η2 = 0.41]. Univariate ANOVA revealed that self-worth was significantly less influenced by others' approval [ F(2,206) = 40.58, p<0.0001, η2 = 0.25] with large effect sizes at both program conclusion and follow-up. They also reported a reduced impact of appearance on self-worth at program conclusion [F(2,206) = 4.0, p<0.02, η2 = 0.03 (Table 6). The improvement maintained at follow-up. Nevertheless, self-esteem, (measured by Rosenberg scale), did not reveal any change between assessment times [(F(2,210) = 1.7, NS].

### Intervention vs. control

At baseline, there were no significant differences between the intervention and the control group in measurements. Over time, mixed results were found in measurement differences between the intervention and the control groups. The means, standard deviations of the changes in measures and the results of the 2×3 (group X time) ANOVA are presented in Table 7.

**Table 7:** ANOVA analysis of the differences between intervention and control groups.

Measures	Change in Intervention Group			Change in Control Group			ANOVA		
	(N = 210)			(N = 49)			(Group X Time)		
	Baseline	Conclusion	Follow-up	Baseline	Conclusion	Follow-up	F	P	η2
Influenced by media	1.85±0.8	2.14±0.8	2.00±0.9	2.38±0.7	2.38±0.8	2.3±0.09	0.83	NS	
Awareness to lifeburdens	1.9±0.09	2.92±0.9	2.81±1.0	2.00±0.7	2.10±0.9	2.14±0.9	6.55	0.01	
Life seems more interesting	1.76±0.7	1.97±0.8	1.98±0.9	1.69±0.9	1.65±0.7	1.70±0.9	3.55	0.02	
Improvement in positive and negative communication	3.63±1.5	3.83±1.6	3.84±1.8	1.77±±1.8	1.05±0.2	0.94±0.2	4.9	0.02	0.04
Number of media strategic identified	4.75±1.38	5.24±1.3	5.0±1.3	4.69±1.5	4.76±1.32	4.58±1.1	5.05	0.02	0.032
Desire to fix appearance	3.35±0.4	3.28±0.7	3.19±0.8	3.19±0.8	3.23±0.7	3.4±0.6	3.53	0.04	
Drive for thinness	8.65±1.3	7.25±1.3	7.33±1.2	6.45±1.5	5.8±1.6	7.7±1.6	4.1	0.03	0.036
Self-worth contingent upon others approval	2.59±0.6	2.05±0.5	2.09±0.5	2.25±0.4	2.75±0.44	2.42±0.5	18.35	0.000	**0.23**
Self-worth contingent upon appearance	2.30±0.5	2.14±0.5	2.19±0.5	1.85±0.4	2.06±0.60	1.97±0.6	3.0	0.047	0.02

Note: Partial η2<0.05 is a small effect, 0.059–0.1 a medium effect and **above 0.1 large effect**. Means, standard deviations.

Significant interaction effects of group X time were found in respect to: awareness of adolescents to life complexity; improvement in use of positive and negative communication, ability to identify media strategies, decrease desire to fix appearance due to media influence, decrease in drive for thinness as well as in self-worth contingent upon others approvals and upon appearance. A large effect size (>0.1) have been demonstrated both in adolescents' awareness to complexity of life and in reduced self-worth contingent upon others approval (Table 7). Change in self-esteem, eating disorders features measured by EAT-26 and body dissatisfaction did not show group X time interactions.

### Influence of participants' self-esteem on program impact

Since self-esteem is considered a risk factor for hazardous behaviors, a one way MANOVA analysis was performed with repeated measures (time) and self-esteem as the independent variable, in order to examine if the program has a different influence on those with low self-esteem compared to those with high self-esteem at baseline. The analysis showed that those with higher self-esteem at baseline, reported greater improvement with respect to awareness of media pressure (F(1,188) = 8.72, p = 0.004; Partial η2 0.09), and showed greater reduction in influence of appearance on self-worth (F(1,181) = 6.07; p = 0.016; Partial η2 0.07).

## Discussion

The current study aimed to evaluate the efficacy of *In Favor of Myself*, a widely disseminated media literacy and dissonance health education interactive universal program. The program aimed to promote positive attitude towards growing up, sense of self and self-esteem, body image and communication among adolescents. The program also aimed to promote the ability to filter external, media and culturally inappropriate messages about diversity of beauty and attractiveness.

At post-test, the intervention group presented with a statistically significant advantage compared with the control group for awareness to changes during adolescence, recognizing usage of media strategies, usage of positive vs. negative communication, self-worth contingent is less relied on others approval and appearance, and lower drive for thinness and desire to fix appearance. An important outcome is the evidence that the program did not reveal any iatrogenic effect for the intervention group. No changes in the intervention group or differences between the groups were found for self-esteem, body dissatisfaction or eating attitudes and behaviors (measured by EAT-26). The lack of changes in these variables reflects the baseline status of our research population. When comparing the mean values of EAT-26 and the EDI-2 body dissatisfaction subscale to those published by others [23]–[25], this study population had much lower scores at baseline in the EAT-26 and in EDI-2 subscales, which may indicate that although they were from 4 different demographic areas, this population was relatively protected from disordered eating at their age (mean 13.5). In addition, this study was performed in less rural areas of the country which might partially explain the low baseline EAT and EDI scores. This explanation is supported by the values reported with respect to body figure perception. The mean current body figure perception was very close to the healthy perception and was not statistically different from the ideal figure. Shaw et al [20] suggested that programs with participants older than 15 years, (our mean age is 13.5 yrs), have greater effects than programs with participants younger than 15, since programs are more effective when implemented during the peak risk period for the emergence of eating disorder symptoms, which has been identified as between 15 and 19 years, by prospective studies. Younger individuals may also have less insight and less ability to apply the principles and skills learned throughout the program, since they are still developing abstract reasoning skills. In addition, lower levels of eating disorder symptoms in younger individuals may lead to lower effect sizes. Moreover, changes in body image are more related to girls than boys and at an older age than the population in this study. Nevertheless, the intervention succeeded in decreasing the gap between current and ideal body figure of girls in the intervention group, thus, it may explain the improvement in drive for thinness.

Support for the effectiveness of prevention intervention strategies designed either to prevent or ameliorate body dissatisfaction or reduce risk factors for eating disorders, is very mixed. Recently, González et al [26] reported that a media literacy-based universal program, delivered to mixed-gender groups, is an effective intervention to reduce long-term, self-reported disordered eating attitudes and internalization of the aesthetic body ideal. Only 15 of 66 eating disorder prevention programs (23%), evaluated in controlled tests, produced significant reductions in current or future symptoms of eating disorders [21], and the effects persisted, after a minimum of 1 year of follow-up, in only three of them (5%). Odea & Abraham [23] also demonstrated that an interactive educational program, aimed at improving self-esteem, can improve drive for thinness. In contrast to our study, these authors succeeded in improving body image and body dissatisfaction as well as self-esteem, while others reported that effect on risk factors for body dissatisfaction, body image and eating disorder symptoms occurs less consistently. For instance, the intervention described by McVey et al. [27] appeared to have an impact on internalization of media ideal for both boys and girls, but the impact on disordered eating for girls occurred only post-intervention. In addition, reduction in weight-loss behaviors was not maintained at follow-up and the program did not have any impact on body satisfaction. In their nine-session intervention, Wick et al [28] improved knowledge and body self-esteem, but their study did not appear to impact eating behaviors [28].

The failure to demonstrate change in self-esteem is not well understood, mainly in light of the high correlation between self-esteem and self-worth contingent upon others' approval and appearance. Still, those with higher self esteem at baseline, reported a greater improvement with respect to awareness of media pressure and showed a greater reduction in influence of appearance on self-worth, which might give some indication about the suitability of the intervention for this population. For example, it may be that participants with low self-esteem need more intensive intervention.

### Limitations and Strengths

A number of limitations in this study must be acknowledged. First, the results must be interpreted with caution, as randomized allocation to intervention and treatment groups was not possible due to timetable conflicts imposed by the school. The lack of random assignment to group conditions might reduce the external validity of this study. Second, the control group was smaller than the intervention group. The research group was balanced with respect to the proportion of girls and boys (55% girls and 45% boys) but the control group was not. (67% girls and 33% boys). This difference might have introduced bias in the comparison between the research and control groups. Third, in this study we used self-report questionnaire thus the answers are subjective and conclusions must be drawn cautiously. In addition, owing to limited resources, longer-term follow-up with the current population was not possible.

Limitations should be considered in the context of the strengths of the study, including the low attrition rates and the high response rate over the three assessment points (82%). Although 256 adolescents filled out the baseline questionnaire, 210 completed the questionnaire during all three-assessment times. Those who did not complete them all, did not drop-out but rather missed school that day or simply did not feel like completing the forms.

More strengths of the program are the use of multiple tools to assess change in perceptions, the examination of both negative and positive outcomes and the use of follow-up assessment to examine the medium-term effects of the intervention. The study included all 3 components of evaluation: process, impact and outcome on knowledge; targeted risk and protective factors, as well as resulting behavioral effects.

To increase the chances of the intervention producing a positive effect and a more consistent impact, the program targets multiple risk factors. (Media images, stereotypes, interpersonal communication, peer influence, media literacy and critical perspective towards the ideal self and body image).

This study provides **initial indication** that In favor of myself may produce effective impact on promoting healthy self- perception. It has a well- established manual and a structured training and supervision program for those who deliver it. The program includes inherent observational and other methods to verify and ensure fidelity in its implementation. The participatory approach increases program generalizability, as well as leaders' sense of ownership. It increases incentive and motivation to continue, because of the vested interest in its success and effectiveness. The program can serve either as a universal or as selective program to promote adolescents' physical and emotional well-being.

### Future Directions

Given that these promising findings are preliminary, future research is needed and should include greater numbers of ethnically diverse individuals, use of random assignment to both intervention and control groups and plan a longer follow-up. Future research may also benefit from use of extensive qualitative data in the form of semi-structured in-depth interviews. Such data have the potential to reveal much about the subjective impressions of the program with regard to both process and outcomes.

To increase the program's impact, it is suggested that this program be applied with ongoing training, professional development of teachers and feedback from evaluation. Moreover, repetition of its topics every year, (if it is delivered via school-based setting), may be suggested in order to address the developmental stage of youth and build a solid foundation of health promotion activities to increase the sustainability of the intervention.
